# Telemedizin in der Rheumatologie

**DOI:** 10.1007/s00393-020-00912-1

**Published:** 2020-10-15

**Authors:** M. Krusche, F. Mühlensiepen, P. Aries, M. Welcker, J. Knitza

**Affiliations:** 1 Medizinische Klinik mit Schwerpunkt Rheumatologie und Klinische Immunologie, Charité Universitätsmedizin, Charitéplatz 1, 10117 Berlin, Deutschland; 2 Zentrum für Versorgungsforschung Brandenburg, Medizinische Hochschule Brandenburg Theodor Fontane, Neuruppin, Deutschland; 3 Fakultät für Gesundheitswissenschaften Brandenburg, Potsdam, Deutschland; 4 Rheumatologie im Struenseehaus, Hamburg, Deutschland; 5 MVZ für Rheumatologie Dr. Martin Welcker GmbH & RheumaDatenRhePort (RhaDaR), Planegg, Deutschland; 6 Medizinische Klinik 3 – Rheumatologie und Immunologie, Universitätsklinikum Erlangen, Friedrich-Alexander-Universität Erlangen-Nürnberg (FAU), Erlangen, Deutschland

**Keywords:** Telefonsprechstunde, Videosprechstunde, Apps, Wearables, Digitalisierung, Telephone consultation, Video consultation, Apps, Wearables, Digitalization

## Abstract

Der Ausbruch der COVID-19-Pandemie geht mit tief greifenden Einschnitten im Alltag und im Berufsleben einher – sowohl gesamtgesellschaftlich als auch speziell im Gesundheitswesen. Im Fokus der Pandemieeindämmung haben sich vielerorts rheumatologische Routineabläufe verändert. Um den entsprechenden Infektionsschutz der Patienten und des medizinischen Personals gewährleisten zu können, wurde hier verstärkt Telemedizin (insbesondere Telefon- und Videosprechstunde) eingesetzt. Weiterhin stehen durch die Digitale-Gesundheitsanwendungen-Verordnung (DiGAV) voraussichtlich in den kommenden Monaten neue, abrechnungsfähige telemedizinische Anwendungsmöglichkeiten wie Apps und Wearables zur Verfügung. Der Artikel soll einen Überblick über telemedizinische Versorgungsmöglichkeiten in der Rheumatologie (mit besonderem Fokus auf die Videosprechstunde) geben. Weiterhin wird Bezug auf die vorhandene Evidenzlage sowie Chancen und Limitation der Telemedizin im Fachgebiet genommen.

## Abstract (en)

Due to the outbreak of the COVID-19 pandemic, in recent months we have experienced deep changes in our daily and professional lives. In the context of pandemic containment, routine rheumatological procedures have changed in many areas. To guarantee sufficient protection against the infection for patients and staff, telemedicine (especially telephone and video consultation) has increasingly been used. Due to the Digital Healthcare Act (DiGAV), whereby patients will have a legal claim to specific digital health applications in Germany, medical apps and wearables will offer new opportunities for telemedical monitoring. This article provides an overview of telemedical care options in the field of rheumatology. Furthermore, opportunities and limitations of telemedicine in rheumatology are reviewed.

## Definition

Telemedizin ist ein Sammelbegriff für ärztliche Versorgungskonzepte, die medizinische Leistungen in den Bereichen Diagnostik, Therapie und Rehabilitation sowie bei der ärztlichen Entscheidungsberatung über räumliche Entfernungen (oder zeitlichen Versatz) mithilfe von Informations- und Kommunikationstechnologien erbringen [1].

## Einleitung

Der Einsatz von Telemedizin hat in den letzten Jahren bereits an Bedeutung gewonnen. Insbesondere in der Kardiologie [2, 3] und Neurologie [4] wurden die Potenziale der Telemedizin mithilfe mehrerer Studien und Projekte belegt. Einen Überblick über die vielfältigen Projekte aus den verschiedenen Fachrichtungen gibt das Deutsche Telemedizin-Portal [5].

**Figure 1:**
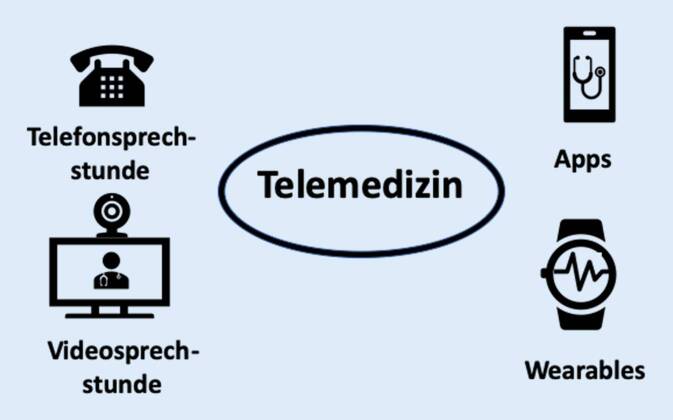

Auch in der Rheumatologie bieten telemedizinische Ansätze neue und spannende Anwendungsmöglichkeiten. Das Fachgebiet beschäftigt sich hauptsächlich mit der Versorgung chronisch kranker Patienten, die größtenteils ambulant-elektiv betreut werden können, weswegen telemedizinische Sprechstunden für ausgewählte Patientengruppen eine sinnvolle Ergänzung zur Routineversorgung darstellen können. Des Weiteren entstehen durch die DiGAV neue, abrechnungsfähige telemedizinische Anwendungsmöglichkeiten (wie z. B. durch die Nutzung von Apps und Wearables) (Abb. 1). Der Artikel gibt einen Überblick über bestehende telemedizinische Versorgungsmöglichkeiten. Weiterhin wird die aktuelle wissenschaftliche Evidenzlage der Telemedizin in der Rheumatologie beleuchtet, sowie Chancen und Limitation der Technik werden erläutert.

## Telefonsprechstunde

Die telefonische Betreuung von Patienten ist die bisher am häufigsten etablierte Form von Telemedizin in der Rheumatologie [6].

Die Voraussetzungen für eine Telefonsprechstunde sind niedrig. Über das Telefon lassen sich einfach und niederschwellig Informationen über den allgemeinen Krankheitszustand, neue Symptome oder organisatorische Informationen (z. B. zur Medikamenteneinnahme oder Terminvereinbarung) sowie Befunde übermitteln.

Neben der verbalen Kommunikation sind hier jedoch keine weiteren Untersuchungen möglich. Nonverbale Kommunikation, ein visueller Eindruck vom Patienten oder die Durchführung von Untersuchungstests kann über das Telefon nicht erfolgen.

Außer im Bereich der PKV (Ziffern 1 und 3) und unter bestimmten Bedingungen in der hausärztlichen Medizin (Ziffer 01435) wird diese Form der sonstigen fachärztlichen Beratung finanziell bisher nicht adäquat vergütet [7].

Telefonsprechstunden können im Hinblick auf bestimmte Fragestellungen auch von geschultem Fachpersonal durchgeführt werden. Studien aus dem Ausland legen außerdem einen zusätzlichen Nutzen von telefonischen Angeboten nahe [8–10].

Bezüglich der Akzeptanz durch die Patienten zeigte eine aktuelle Studie aus Spanien jedoch gemischte Ergebnisse. Nur 52,7 % der 220 in einer Studie befragten Patienten, die eine Telefonkonsultation erhielten, bewerteten diese als nützlich [11].

Im Rahmen von Interventionsstudien wurde der Nutzen von telefonischen Betreuungsprogrammen bereits nachgewiesen. Hier wurde z. B. bei Gichtpatienten gezeigt, dass mittels regelmäßiger telefonischer Interventionen durch eine Krankenschwester die Serumharnsäurewerte sicher und effektiv gesenkt werden konnten [12].

Eine amerikanische Studie zeigte, dass mithilfe von elektronischen Visiten (E-Visits) bei Gichtpatienten eine signifikante Senkung der Serumharnsäurekonzentrationen möglich war. In dem Programm erhielten die Patienten elektronische Benachrichtigungen und Informationsmaterial. Außerdem erfolgte eine regelmäßige Abfrage der Harnsäurewerte. Die Ergebnisse wurden ärztlich beurteilt, und die Patienten erhielten ein elektronisches oder telefonisches Feedback vom Arzt [13].

Gängige Praxis in der Routine ist, dass via Telefon eine erste Triagierung der Akutpatienten (meist durch geschultes Fachpersonal) erfolgt. Bei bereits bekannten (weniger komplexen) Patienten kann die Telefonsprechstunde bei stabilem Verlauf durchaus ergänzend nützlich sein und ggf. auch temporär überbrückend eingesetzt werden. Weiterhin ermöglichen Telefoninterventionen die Steigerung der Therapieadhärenz der Patienten.

## Videosprechstunde

**Figure 2:**
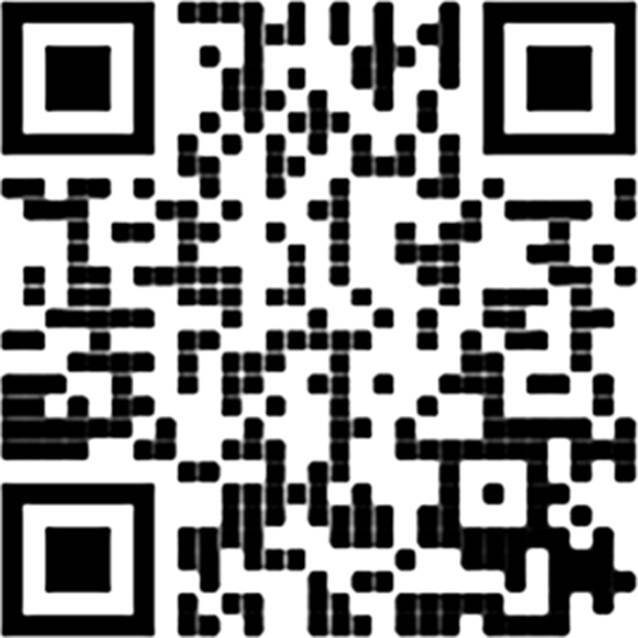

Insbesondere während der COVID-19-Pandemie hat die Videosprechstunde vermehrt an Zulauf gewonnen, weswegen ihr der vorliegende Artikel besondere Beachtung schenkt. Für die Videosprechstunde wurde im Zuge der aktuellen medizinischen Notfallsituation in Deutschland sogar vorübergehend eine unbegrenzte Nutzung erwirkt und die Leistungserstattung verbessert. Zudem ist mittlerweile sogar die Krankschreibung per Videosprechstunde möglich [14]. Neben dem Austausch von verbalen Informationen können Arzt und Patient visuell miteinander interagieren. Somit können pathologische Haut- und Gelenkbefunde und die aktive Beweglichkeit einiger Gelenke (insbesondere der oberen Extremität) demonstriert werden. Demonstrationsvideos für die praktische Durchführung bietet u. a. Jack Cush im Internet an (Abb. 2; [15]).

## Praktische Umsetzung

Die Umsetzung einer Videosprechstunde erfordert die Registrierung bei einem zertifizierten Videodienstanbieter. Einen Überblick über die aktuell zertifizierten Videodienstanbieter bietet u. a. die Homepage des Health Innovation Hub (hih) des Bundesministeriums für Gesundheit [16].

Wenngleich die technischen Voraussetzungen der Videokommunikation im Alltag, insbesondere seit Ausbruch der Corona-Pandemie, meist gegeben sind, stellen diese im medizinischen Setting keine Selbstverständlichkeit dar. Es wird eine von beiden Seiten stabile Internetverbindung benötigt. Die Ton- und Bildqualität muss beidseits störungsfrei sein, und eine datensichere Kommunikation ohne wiederholte Störung (z. B. durch Praxismitarbeiter oder auch Familienangehörige) muss sichergestellt sein.

**Figure 3:**
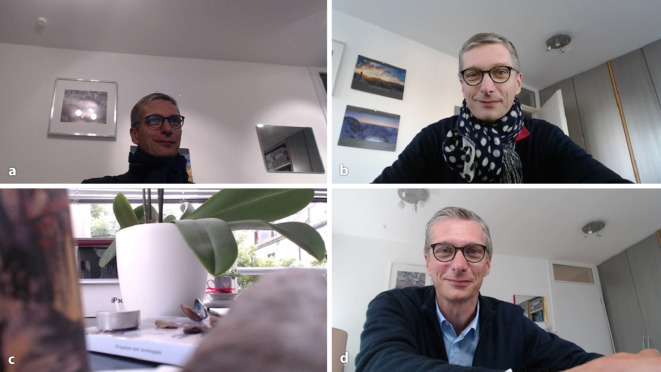

Für die erfolgreiche Durchführung der Videosprechstunde ist insbesondere eine gute Vorbereitung notwendig. Die technische Durchführung sollte schon mehrmals vonseiten der Ärzte erprobt worden sein, um immer wieder auftretende technische Unzulänglichkeiten kurzfristig beheben zu können. Dazu gehört die Prüfung der Bild- und Tonqualität. So sind hallende Räume, Gegenlicht durch Fenster oder Lampen, zu tiefe Kameraposition, ungeeignete Kleidung und z. B. unruhige Bildhintergründe schlecht, nachteilig für die Qualität der Videosprechstunde (vgl. Abb. 3). Zudem muss das Procedere der Einladung der Patienten innerhalb der Institution bzw. Praxis klar geregelt sein. Dieses muss nicht durch die Ärzte selber erfolgen, sondern kann delegiert werden. Zusammen mit der Einladung an die Patienten über E‑Mail oder SMS sollte eine kurze Anleitung mit gesendet werden, wie und wann sich die Patienten in dem virtuellen Warteraum der Videosprechstunde einwählen sollen [17].

## Durchführung, Zeitmanagement und Dokumentation

In der Praxis haben sich 2 unterschiedliche Systeme bei der Durchführung der Videosprechstunde etabliert. Zum einen kann im Laufe des Tages ein Zeitkorridor für die Durchführung aller Videosprechstunden angeboten werden. In diesem Zeitkorridor wird entweder eine Terminsprechstunde oder aber auch eine offene Sprechstunde angeboten. Eine andere Möglichkeit ist es, die Videosprechstunde statt einer Präsenzsprechstunde zum gleichen Zeitpunkt durchzuführen, sodass die Terminplanung bestehen bleibt, nur zwischen virtueller und Präsenzsprechstunde gewechselt wird. Erfahrungsgemäß warten Patienten virtuell jedoch weniger lang im digitalen Wartezimmer als in der Praxis. Nach Durchführung der Videosprechstunde ist, wie auch bei jedem anderen Kontakt mit dem Patienten, eine entsprechende Dokumentation im Praxisverwaltungssystem bzw. Krankenhausinformationssystem notwendig. Für das Ansetzen der Abrechnungsziffern sind im kassenärztlichen System auch das Schreiben und Versenden eines Arztbriefes obligater Inhalt der Leistungsziffern. Im Rahmen der gesicherten Verbindung, welche Voraussetzung für die Zulassung des Videoportals seitens der KBV ist, können aber bei entsprechender technischer Ausstattung auch Dokumente (z. B. Röntgenbefunde) ausgetauscht, dargestellt bzw. besprochen werden.

## Datenlage

In einer amerikanischen Übersichtsarbeit zum Einsatz von verschiedenartigen Telemedizininterventionen in der Diagnose und Behandlung von rheumatischen Erkrankungen wurden 20 Studien identifiziert und ausgewertet [18]. Zwölf von 20 Studien untersuchten dabei den Einsatz von Videosprechstunden. Insgesamt bewerteten in der Analyse 10 von 12 Studien den Einsatz von Videosprechstunden als effektiv. Sieben Studien verwiesen auf hohe Zufriedenheitswerte bei Patienten und Versorgern. Vier Studien hoben Kosten- bzw. Zeitersparnisse hervor. Eine weitere, ältere Studie aus dem Jahr 2000 bewertete den Einsatz von Videosprechstunden als ineffektiv. Dies wurde mit der niedrigen Bildqualität der genutzten Kamera und verbundenen Diagnoseungenauigkeiten begründet [19].

Die einzige inkludierte randomisiert kontrollierte Studie (RCT) zum Einsatz von Videosprechstunden in der Versorgung von Patienten mit rheumatoider Arthritis (RA) berichtete keinen qualitativen Unterschied zwischen Videokonferenz und persönlicher Vorstellung, bezogen auf DAS28-CRP, RADAI, mHAQ und EQ5D [20]. McDougall et al. schlussfolgern, dass die Studienlage einen positiven Eindruck von Telemedizin in der Rheumatologie vermittelt. Die Evidenz ist jedoch begrenzt, und randomisierte Studien sowie Analysen zur Kosteneffektivität fehlen, um die Kontextfaktoren zum effektiven Einsatz von Telemedizin abschließend bewerten zu können [21].

Eine weitere RCT legt die Effektivität eines Patient-Reported-Outcome(PRO)-basierten telemedizinischen Interventionsprogramms in der Versorgung von RA-Patienten mit geringer Krankheitsaktivität oder Remission nahe [22]. Demnach unterschied sich der Krankheitszustand der Patienten in telemedizinischer Nachsorge nicht von dem Krankheitszustand der Patienten in Standardversorgung. Interessanterweise wurde in der Studie kein Unterschied zwischen der telemedizinischen Versorgung durch Ärzte oder Fachassistenten/innen festgestellt.

**Figure 4:**
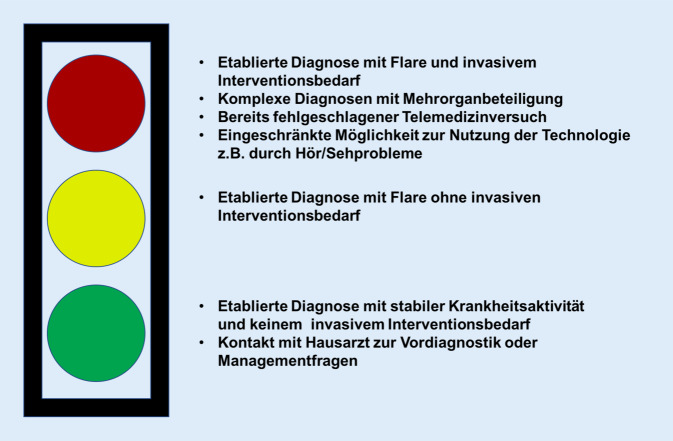

Dass eine Videosprechstunde jedoch nicht für alle Patienten geeignet ist, legt die Arbeit von Kulcsar et al. nahe, in der 19 % der Patienten als nicht Telerheumatologie-fähig beurteilt wurden [23]. Die hierfür genannten Gründe waren, dass die Grunderkrankung entweder zu unklar oder die Erkrankung zu komplex seien. (In Anlehnung an die Publikation von Kulscar et al. schlagen die Autoren für die Triagierung der Patienten einen modifizierten Algorithmus vor [vgl. hierzu Abb. 4]).

Insgesamt zeigt sich, dass die Datenlage zum Einsatz von Videosprechstunden in der Rheumatologie bisher sehr begrenzt ist und die untersuchten Patientengruppen heterogen sind. Positive Effekte wurden in einzelnen Studien gezeigt, jedoch gilt auch festzuhalten, dass einige Patienten aufgrund der Komplexität der Grunderkrankungen, mangelnden Technikzugangs oder der beschränkten Digitalkompetenzen ungeeignet für die Videosprechstunde sind. Die Studienlage, bei welchen Patienten die Videosprechstunde tatsächlich zu keinem Qualitätsverlust in der Versorgung führt, muss daher durch weitere, möglichst prospektive Untersuchungen vor deren routinemäßigem Einsatz verbessert werden.

## Apps und Wearables

Durch die DiGAV können Ärzte medizinische Apps verordnen. Ein großer Vorteil von Apps (mobilen Applikationen) und Wearables (unmittelbar am Körper getragene Sensoren, wie z. B. Schrittzähler oder die Apple Watch™ [Cupertino, USA]) besteht die Möglichkeit der bequemen asynchronen Kommunikationsmöglichkeit zwischen Arzt/RFA und Patient.

Prinzipiell unterscheiden kann man zwischen der aktiven und passiven digitalen Datenerfassung. PROs wie der RAPID‑3 [24] eignen sich gut zur kontinuierlichen digitalen Therapieüberwachung beispielsweise mittels wöchentlicher Erinnerung und Eingabe per App [22]. Ebenfalls gibt es bereits Pilotstudien zur Erfassung von Gelenkschwellungen per Smartphonekamera [25] und Handkraftmessung [26] per App.

Die App-Eingabe sollte optimaler Weise 1‑mal wöchentlich stattfinden und nicht länger als 15 min dauern [27]. Die aktuelle Bereitschaft von deutschen Rheumatologen, entsprechende elektronische PROs (ePRO) regelmäßig zu sichten, ist bisher jedoch eher gering [28].

Noch bequemer für Patienten ist die passive Datenerfassung mittels objektiven digitalen Biomarkern. Hier steckt die Rheumatologie noch in den Kinderschuhen. In einer Pilotstudie konnten durch Schrittzahlmessung präzise Vorhersagen von Krankheitsschüben bei Patienten mit rheumatoider Arthritis und axialer Spondyloarthritis gemacht werden [29]. Auch bei selteneren Erkrankungen wie den inflammatorischen Myopathien ermöglichen sensorbasierte Daten Rückschlüsse auf Krankheitsaktivität und Therapieansprechen [30].

Neben der reinen Datenerfassung kann über digitale Erinnerungsfunktionen oder Edukationsprogramme auch die Therapieadhärenz und das Krankheitsmanagement verbessert werden. So zeigte u. a. die Pilotstudie von Mary et al. eine Verbesserung der Therapieadhärenz für die Einnahme von Methotrexat durch den Einsatz von SMS-Erinnerungen bei RA-Patienten [31].

Eine amerikanische Pilotstudie an RA- und Lupus-Patienten zeigte, dass Patienten, die mittels Fitbit™ (San Francisco, USA) ihre physische Aktivität monitorierten und parallel telefonische Follow-up-Anrufe von Physiotherapeuten erhielten, sich nach 27 Wochen signifikant mehr bewegten und weniger Schmerzen haben [32].

Apps und Wearables für medizinische Zwecke sind aktuell (auch aufgrund der DiGAV) ein neues und viel umworbenes Feld, das von verschiedenen Akteuren genutzt wird [33]. Die Einsatzmöglichkeiten dieser Technologien z. B. im Rahmen eines kontinuierlichen Krankheitsmonitorings oder zur Patientenedukation sind vielfältig [34]. Die aktuelle Studienlage zum Nutzennachweis ist momentan jedoch noch überschaubar und insbesondere der Datenschutz bei einigen Applikationen aktuell noch als kritisch zu bewerten [35, 36].

## Limitationen von Telemedizin

Trotz der vielseitigen Einsatzmöglichkeiten von Telemedizin ergeben sich spezifische Nachteile und Grenzen von telemedizinischen Ansätzen in der rheumatologischen Versorgung.

Gleichzeitiger Vor- und Nachteil der Telemedizin ist der fehlende direkte und persönliche Kontakt zwischen Patienten und Arzt. Hieraus ergeben sich verschiedenartige Konsequenzen für die Patientenbehandlung [37, 38].

### Untersuchungs- und Kommunikationsprobleme

Die Behandlung per Informations- und Kommunikationstechnologie ermöglicht in der Regel keinen Gesamteindruck des Patienten. Auffälligkeiten im Bewegungsablauf lassen sich nur begrenzt nachvollziehen. Gelenke können vom Arzt selbst nicht palpiert und die Bewegungsgrade bestimmt werden [38]. Ein umfassender körperlicher Status (z. B. Auskultation oder Perkussion) ist nicht möglich, sodass durch die Videosprechstunde viele Untersuchungsbefunde nicht vollumfänglich zu erheben sind. Hierdurch können wichtige Informationen verloren gehen. Insbesondere für den Erstkontakt oder bei komplexen Krankheitsfällen kann die Videosprechstunde keine adäquate Untersuchungsqualität gewährleisten [23, 24].

Zudem verändert sich die Arzt-Patienten-Kommunikation aufgrund der äußeren Rahmenbedingungen. Nonverbale Kommunikation lässt sich viel schwieriger erfassen. Psychosoziale Behandlungsbedürfnisse der Patienten können in der telemedizinischen Versorgung untergehen oder werden vom Patienten gar nicht erst thematisiert [39]. Diese Barrieren werden unter Umständen durch eine niedrige Bild- und Tonqualität verstärkt, sodass behandlungsrelevante Informationen verloren gehen können. Die aktive Beteiligung des Patienten ist stärker gefordert als in der persönlichen Sprechstunde. Eine gute Gesprächsführung ist unerlässlich. Ein unstrukturiertes Vorgehen kann zu Missverständnissen bei Arzt und Patient führen [37].

### Technikkompetenz und Zugang

Ein weiterer wichtiger Aspekt für den effektiven Einsatz von Telemedizin in der Rheumatologie sind die Technikkompetenz der Patienten und Ärzte sowie die Zugriffsmöglichkeiten auf die jeweilige Technologie. In ländlichen und strukturschwachen Regionen kann die Breitbandverfügbarkeit den Einsatz von Telemedizin gänzlich verhindern [40]. Ältere und insbesondere sozial Schwache verfügen, wie auch die COVID-19-Pandemie zeigt, häufig nicht über das entsprechende technische Equipment. Dies hat sich aktuell auch in der Diskussion um die Corona-App gezeigt, da insbesondere ältere Mobiltelefone nicht die notwendige Softwareaktualisierung aufwiesen. Weiterhin sind teilweise sowohl Patienten als auch das medizinische Personal und Ärzte nur unzureichend in der Nutzung entsprechender Technologien geschult [37].

### Besondere Anforderungen an die Praxisabläufe und das Personal

Die Implementierung von Telemedizin in die ambulanten Praxisabläufe erfordert die Aufmerksamkeit des gesamten Behandlungsteams und bindet zeitliche und finanzielle Ressourcen. Dies umfasst die Identifizierung, Anschaffung und Erprobung der adäquaten technischen Lösungen, die Überprüfung des Datenschutzkonzeptes, die Anpassung der Praxisabläufe, Sprechstundenzeiten und unter Umständen der räumlichen Gegebenheiten.

Die potenzielle Datenflut, die durch App-Monitoring oder Wearable-Daten entsteht, muss sinnvoll und sicher gebündelt und nach Möglichkeit digital voranalysiert werden. Hier könnte z. B. eine entsprechend qualifizierte rheumatologische Fachkraft sinnvoll mit eingebunden werden. In einigen Ländern wie den USA, Kanada oder Großbritannien gibt es bereits das Berufsbild der Telenurse [41].

### Vergütung

Weiterhin gilt es, die wirtschaftliche Dimension von Telemedizin sowie Vergütungsmodalitäten, insbesondere für Einzelpraxen, vorab zu prüfen. Die Vergütung von Videosprechstunden wurde in den letzten Jahren kontinuierlich überarbeitet, und bisher geltende Beschränkungen in der Abrechnung von Videosprechstunden im Rahmen der COVID-19-Pandemie, einschließlich des dritten Quartals 2020, wurden aufgehoben. Zudem sind Videosprechstunden bei allen Indikationen erlaubt – auch dann, wenn ein Patient vorher noch nicht in der Praxis war. Es existieren jedoch in der ambulanten Regelversorgung derzeit noch keine klaren Vergütungsstrukturen für telemedizinische Ansätze, die über Telefon- und Videosprechstunden, Telekonsile sowie die Überwachung von Herzschrittmachern hinausreichen [42]. Die Betreuung der Patienten via Videosprechstunde ist, abgesehen von den qualitativen Einschränkungen, aus wirtschaftlichen Gründen bisher meist nicht attraktiv, da die Honorierung bisher die Kosten für Technik und Personal nicht deckt. Zudem genügt die derzeitige ärztliche Kapazität nicht, um die erforderliche Anzahl an Terminen zu generieren. Die zusätzlich notwendigen Zeitfenster sind langfristig nur mit einem Mehr an Ärzten und medizinischem Fachpersonal realisierbar.

### Datenschutz

Ein weiterer wesentlicher Aspekt ist der Datenschutz, der für eine erfolgreiche Implementierung und Akzeptanz von Telemedizin dringend erforderlich ist. Unerlaubtes Mitschneiden oder Filmen der Gespräche ist technisch sehr einfach. Insbesondere hochsensible medizinische Daten und die Interaktion von Patient und Arzt müssen daher entsprechend geschützt werden [43, 44]. Hier ist der Gesetzgeber gefordert, entsprechende Sicherheitsstandards zu definieren und regelmäßig zu überprüfen.

### Evidenz und Vergleichbarkeit

An letzter Stelle verweisen die Autoren auf die begrenzte Evidenz zum Einsatz von Telemedizin in der rheumatologischen Versorgung. Telemedizin ist ein Oberbegriff, unter dem sich viele, oftmals heterogene Interventionen sammeln. Es gibt kaum randomisierte kontrollierte Studiendaten, und die Studien adressieren meist nur Teilbereiche des Gesamtkonzeptes „Telemedizin“, weswegen sie und die darin untersuchten Interventionen bisher nur begrenzt vergleichbar sind.

## Zusammenfassung

Durch den rasanten technischen Fortschritt und die Beschleunigung der Digitalisierung durch die COVID-19-Pandemie bieten sich auch in der Rheumatologie spannende neue Möglichkeiten der Patientenversorgung und des Patientenmonitorings via Telemedizin: Mittels Videosprechstunde können örtlich und zeitlich flexible Visiten bedarfsgerecht im Sinne eines „Tight Control“ abgehalten werden. Regelmäßige automatisierte elektronische oder telefonische Abfragen bezüglich der Medikamenteneinnahme können den Behandlungserfolg bzw. die Therapieadhärenz verbessern [45]. Wearables (wie z. B. Schritt- und Aktivitätsmesser) oder die Nutzung von ePROs können die Erkennung von Krankheitsflares vereinfachen. Weiterhin können Patienten über Apps oder Wearables zu mehr körperlicher Aktivität animiert werden.

Im Zuge der COVID-19-Pandemie und des Infektionsschutzes sind telemedizinische Versorgungsmöglichkeiten auch im Hinblick auf das „Social Distancing“ noch einmal stärker in den Fokus gerückt [46]. Insbesondere die Videosprechstunde hat vielerorts einen rasanten Zulauf erhalten und wird für bestimmte Patientengruppen sicherlich auch in der Post-Corona-Zeit eine sinnvolle Alternative darstellen.

Nichtsdestotrotz bleibt festzuhalten, dass die Telemedizin in absehbarer Zeit den traditionellen Arztbesuch nicht vollumfänglich ersetzen kann und auch nur für selektierte Patientenkollektive in bestimmten Situationen sinnvoll ist. Die Erstvorstellung von Patienten, komplexe Krankheitsfälle oder nicht adäquat eingestellte Krankheitsverläufe müssen weiterhin physisch von einem Arzt gesehen, untersucht und behandelt werden. Weiterhin sind die Digitalkompetenz und der Zugang zu entsprechender Technik in einigen Fällen sowohl für die Patienten als auch die Ärzte entscheidende limitierende Faktoren. Hier wird man in der Zukunft sowohl in der medizinischen Ausbildung als auch auf entsprechende Patientenschulungen einen Fokus legen müssen.

Rahmenbedingungen für einen sicheren Datenschutz, eine flächendeckende leistungsfähige Internetabdeckung sowie v. a. auch adäquate Vergütungsmodelle sind Themen, die die Politik adressieren muss, damit Telemedizin erfolgreich zum Einsatz kommen kann.

Nach wie vor besteht ein rheumatologischer Versorgungsmangel in Deutschland, der sich auch in absehbarer Zeit nicht verbessern wird [47]. Prinzipiell haben die Digitalisierung und die Telemedizin das Potenzial, ortsunabhängig Versorgungsdefizite (insbesondere in unterversorgten Regionen) teilweise zu kompensieren. Allerdings muss die Digitalisierung dabei helfen, Prozesse zu erleichtern. Es darf durch sie kein zusätzlicher Mehraufwand für das Behandlungsteam entstehen.

Zusammenfassend lässt sich festhalten, dass Telemedizin das Potenzial hat, den Zugang zu einer adäquaten Versorgung zu beschleunigen und zu verbessern. Weiterhin kann Telemedizin die rheumatologischen Behandlungsprozesse in bestimmten Settings unterstützen. Wichtig hierfür sind insbesondere die Auswahl der richtigen Patienten in der richtigen Behandlungssituation und eine gute Digitalkompetenz sowohl von Arzt als auch Patient. Damit dieses Potenzial ausgeschöpft werden kann, müssen entsprechende infrastrukturelle Voraussetzungen (IT-Infrastruktur und Sicherheit) sowie eine angemessene Vergütung gewährleistet werden.

## Interessenkonflikt

F. Mühlensiepen gibt die Förderung eines wissenschaftlichen Projektes der Telemedizin durch die Firma Novartis Pharma GmbH an. M. Welcker gibt die Förderung eines wissenschaftlichen Projektes der Telemedizin durch die Firma AbbVie Pharma GmbH und themenbezogene Vortragshonorare der Firmen UCB, AbbVie, Lilly, Sandoz und Novartis an. J. Knitza gibt die Förderung wissenschaftlicher Projekte der Telemedizin durch die Firma Novartis Pharma GmbH und UCB Pharma GmbH an. M. Krusche und P. Aries geben an, dass kein Interessenkonflikt besteht.
